# Development and Validation of a Food Frequency Questionnaire for Evaluating the Nutritional Status of Patients with Cancer

**DOI:** 10.3390/nu15041009

**Published:** 2023-02-17

**Authors:** Se-A Lee, Hyo-Kyoung Choi, Seon-Joo Park, Hae-Jeung Lee

**Affiliations:** 1 Department of Food and Nutrition, College of Bionanotechnology, Gachon University, Seongnam-si 13120, Republic of Korea; 2 Korea Food Research Institute, 245, Nongsaengmyeong-ro, Iseo-myeon, Wanju-gun 55365, Republic of Korea; 3 Institute for Aging and Clinical Nutrition Research, Gachon University, Seongnam-si 13120, Republic of Korea; 4 Department of Health Sciences and Technology, GAIHST, Gachon University, Incheon 21999, Republic of Korea

**Keywords:** FFQ, cancer, validation, Korean

## Abstract

Patients with cancer need to maintain proper nutritional status to overcome cancer, alleviate the side effects of chemotherapy, and prevent a recurrence. As such, it is necessary to manage nutritional status. This study aimed to develop a dish-based semi-quantitative food frequency questionnaire (FFQ) to evaluate the nutritional status of patients with cancer and assess the validity of the FFQ. A total of 109 dish items were selected through contribution and variability analyses using the 2016–2018 Korea National Health and Nutrition Examination Survey data. The FFQ was validated against the average 3-day dietary records of 100 patients with cancer. Pearson correlation coefficients and quartile agreements between FFQ and 3-day dietary records were calculated for intake of energy, macronutrients, and micronutrients. Age and energy-adjusted Pearson correlation coefficients ranged from 0.20 (iron) to 0.54 (potassium). The percentage of participants who were classified into the same or adjacent quartile between the FFQ and the 3-day dietary record ranged from 68% (protein) to 81% (energy, dietary fiber). The results suggest that the FFQ is an appropriate tool for assessing nutritional status in Korean cancer patients.

## 1. Introduction

According to the 2019 National Cancer Registration Statistics of the Korea National Cancer Center, the number of cancer cases among Koreans in 2019 was 254,718, an increase of 3.6% from 2018 [1]. Over the last decades, the Korean diet has changed from a rice-based traditional diet to a Western diet rich in fat and protein [2]. These changes in dietary patterns could cause chronic diseases such as obesity and damage the function of regulating carcinogenesis in the body, which could lead to an increase in the incidence of cancer [2,3].

Dietary factors are known to reduce the risk of cancer and suppress cancer prognosis [4]. The European Code Against Cancer 4th Edition, released by the International Agency for Research on Cancer (IARC), reported that healthy diets are a critical part of cancer patients’ care before and after treatment, and can help prevent common side effects of cancer treatments such as weight loss, palate change, and loss of appetite [5]. Healthy diets include consuming enough grains, legumes, vegetables, and fruits, restricting red meat and high-calorie and salty foods, and avoiding processed meats and sugary drinks [6].

The National Cancer Center of the Republic of Korea reported that diet-related cancers including stomach, colorectal, and breast cancer are among the most common cancers in Koreans, and that dietary factors related to cancer include fruits, vegetables, legumes, and meat [7]. A meta-analysis reported that the consumption of fruits, vegetables, and legumes could reduce the risk of stomach cancer, consumption of high-salt foods could increase the risk of stomach cancer, and elevated meat consumption could increase the risk of colorectal cancer among Koreans [8]. The Korean Multimodal Cancer Cohort study reported that frequent consumption of legumes and tofu was associated with reduced gastric cancer risk in women [9]. A Korean cancer screening examination cohort study found that intake of ≥43 g of red meat per day increased non-gastrointestinal cancer risk in men [10].

It is necessary to develop a well-designed dietary intake assessment tool to identify dietary factors related to cancer [11]. There are various methods for evaluating dietary status, such as the food frequency questionnaire (FFQ), 24 h dietary recall, and dietary records [12]. However, 24 h dietary recall and dietary records have a disadvantage in that they have to be measured repeatedly over time [13] because significant errors occur when measuring long-term intake [14]. However, the FFQ has the advantage of being able to evaluate long-term dietary intake, reducing the burden and expense of participants, and making it easy to understand the relationship between cancer and chronic diseases [12].

As cancer incidence and survival rates in Koreans increase [1], patients with cancer experience a variety of side effects that can lead to serious nutritional damage due to anti-cancer treatment [15]. Therefore, a well-designed FFQ could be used for nutritional management of malnourished patients with cancer suffering from the side effects of chemotherapy.

To date, only two FFQs have been developed for patients with cancer in Korea. A dish-based FFQ was developed by extracting cancer-related dietary factors from the 2001 Korea National Health and Nutrition Examination Survey (KNHANES) and the 2002 Korean National Nutrition Survey by Season [16]. The other FFQ was developed using 3-day dietary records of 192 Korean breast cancer survivors [17]. However, neither FFQ was disclosed, and the FFQ developed by Shin et al. [17] was not validated.

Therefore, this study developed an FFQ to assess the nutritional status of Korean patients with cancer and to evaluate its validity.

## 2. Materials and Methods

### 2.1. Selection of Cancer-Related Dietary Factors

Cancer-related dietary factors (CRDFs) and non-CRDFs were selected to extract a list of dishes in the FFQ. CRDFs are diet-related factors that increase or protect against cancer risk. CRDFs are relevant to cooking methods and ingredients. Given their focus on cooking rather than food, they seemed to be suitable for assessing cancer-related dietary factors in Koreans [16]. Energy, carbohydrates, protein, and fat, which are essential nutrients for calories, were added as non-CRDFs.

To select CRDFs, we reviewed reports from the Korean National Cancer Center, IARC, the World Cancer Research Fund (WCRF), the American Institute for Cancer Research (AICR), and the results of 24 domestic and international cohort and case-control studies. The WCRF and the AICR reported that dairy can reduce the risk of breast and colorectal cancers and that fish can reduce the risk of liver and colorectal cancers. The IARC classified Chinese-style salted fish as a carcinogen, and processed meat as a definite cause of cancer [18]. Therefore, we searched dietary factors associated with cancer in more than one publication, and finally selected nine food groups (red meat, processed meat, dairy products, garlic, whole grains, legumes, alcohol, fish, and vegetables) and three nutrients (vitamin C, Na, and Fe) as the CRDFs. According to food characteristics, fish were divided into three subgroups (fish: whole fish; fish1: whole fish except canned fish; fish2: back blue fish including canned fish) and vegetables were divided into four subgroups (vegetable: whole vegetables; vegetable1: whole vegetables except kimchi; vegetable2: raw vegetables; and vegetable3: kimchi). Finally, a total of 21 dietary factors (DFs) (red meat, processed meat, dairy products, garlic, whole grains, legumes, alcohol, fish, fish1, fish2, vegetables, vegetable1, vegetable2, vitamin C, Na, Fe, energy, carbohydrates, protein, and fat) were used in the analysis.

### 2.2. Selection of Dish Lists

In order to extract dishes related to CRDFs and non-CRDFs, 1279 dishes consumed by participants in the 2016–2018 Korean National Health and Nutrition Examination Survey were used. Contribution analysis (CA) and variability analysis (VA) were performed to select FFQ dish items. CA is a statistical method that calculates the cumulative contribution of a dish to each DF. For example, all dishes can contribute to energy, but CA will sort all dishes by their highest contribution first. When selecting dishes that contributed up to 90% of the 21 DFs, 579 dishes met these criteria. Therefore, we selected dishes until the cumulative ratio of each DF reached 50%. VA is a statistical method that calculates the between-individual variance of dish intake for DF through multivariate regression analysis. VA enabled us to select dish items while considering inter-individual variability. When we selected dishes that contributed up to 90% of the 21 DFs, 438 dishes met these criteria, and 88 dishes contributed up to 50%. Therefore, we included dishes up to the point where the cumulative sum of VA was 70%.

**Figure 1:**
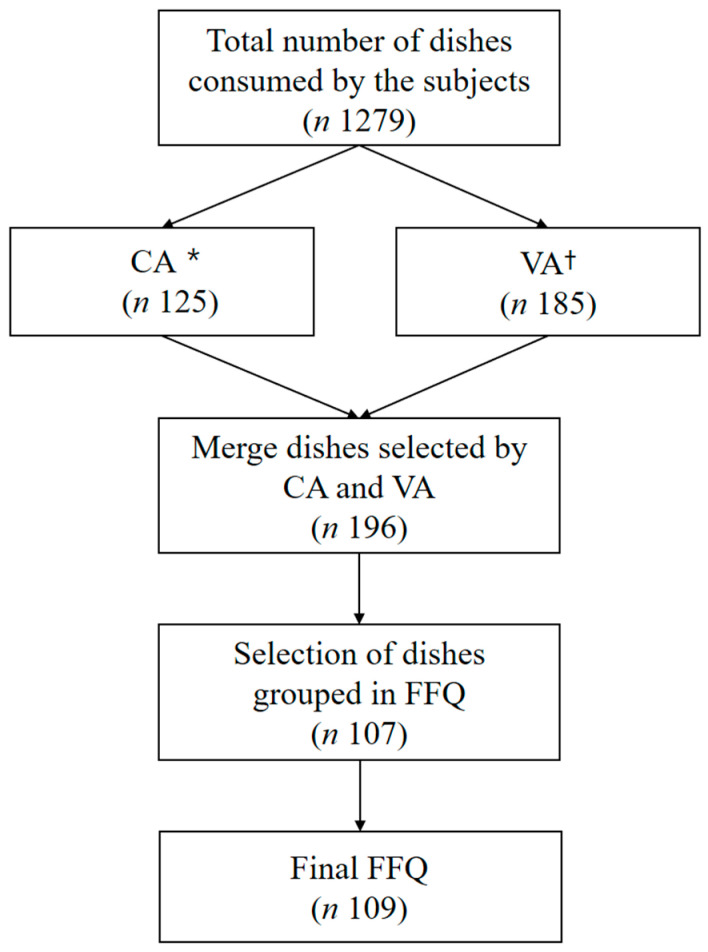
Steps used to develop a list of 109 dishes from the 1279 dishes consumed by the participants. * Contribution analysis (CA) is a statistical method to identify the contribution of dishes to dietary factors. † Variability analysis (VA) is a statistical method to identify inter-individual variability of the dishes to dietary factors.

Of the 1279 dishes eaten by the participants, the final dish lists were selected using the CA and VA methods. A total of 196 dish items were extracted. After regrouping based on the similarity of main ingredients and cooking method, a final 107 dish items were selected. Then, honey and ginger were added to the FFQ list (Figure 1) because they are known to reduce the side effects of anti-cancer treatment. A meta-analysis has reported that honey can enhance the therapeutic efficacy of patients with cancer and delay and reduce the onset of oral mucositis [19,20,21,22]. Ginger is also a traditional vomiting remedy [23] and may reduce nausea, vomiting, and fatigue [24].

### 2.3. Decision of Frequency Response and Portion Size

The frequency of dishes was divided into nine categories (never or rarely, once per month, two to three times per month, once per week, two to four times per week, five to six times per week, once per day, twice per day, and three times per day). For seasonal fruits, the participants responded with 3, 6, 9, and 12 months of intake, frequency, and amount.

Referring to previous studies on FFQ development for cancer patients [16,25], the portion size of each dish was determined based on median intake (weight, volume) of participants in the 2016–2018 KNHANES data, and the portion size identified in CAN-Pro 5.0 (Computer Aided Nutritional Analysis Program 5.0, The Korean Nutrition Society, Seoul, Korea). After determining portion size, those who ate smaller portions were asked to respond with 0.5, and those who ate more were asked to respond with 1.5.

### 2.4. Development of Recipes for Dishes

The recipe database for nutrient calculation for each of the 109 dish items in the FFQ was created using the frequency of each dish in the 2016–2018 KNHANES data. From the KNHANES data, we selected 294 dish items similar to the 109 dish items in the FFQ, and 12,701 ingredients used in the 294 dish items were extracted. Recipes were created considering the contribution and frequency of the extracted 12,701 ingredients, and ingredients with a frequency of less than 1% were removed. Recipes for the 109 dish items consisted of 1828 ingredients.

### 2.5. Validation Study

The FFQ validation study was conducted using a 3-day dietary record through an online program. To validate the FFQ, patients with cancer were recruited from October to November 2021 through online cancer communities. The inclusion criteria of this study were as follows: (1) histologically confirmed cancer; (2) currently receiving chemotherapy or completed chemotherapy within 1 year; (3) adults aged 20 years or older; and (4) active 50% of their waking hours. The exclusion criteria for this study were as follows: (1) pregnant women; (2) individuals with psychiatric problems; and (3) those who could not eat properly due to chemotherapy.

The questionnaire included the following: anthropometric data, dietary habits, lifestyle, disease, drugs, dietary supplements, issues concerning female fertility, family history, anti-cancer drug-induced side effects, FFQ, and 3-day dietary records (two weekdays and one weekend or a holiday). A non-consecutive 3-day dietary record was collected from pre- and post-meal photographs to clarify the amount of food intake. Collected data from the FFQ and 3-day dietary records were reviewed by trained dieticians and checked via social networking service or telephone if corrections or supplements were needed. Nutrient intake calculations from the FFQ and dietary record surveys were performed using CAN-Pro 5.0 (The Korean Nutrition Society).

All procedures involving human participants were approved by the Institutional Review Board of Gachon University School. Written informed consent was obtained from all participants (IRB No. 1044396-202108-HR-182-01).

### 2.6. Statistical Analysis

Means and standard deviations (SDs) of nutrient intake were calculated from FFQ and 3-day dietary records, and nutrient intakes from FFQ and 3-day dietary records were compared using Wilcoxon signed-rank tests. Nutrient data were analyzed after log transformation to improve the normality of the nutrient intake distribution. Pearson’s correlation was used to identify the correlation of unadjusted, energy-adjusted, and energy- and age-adjusted data between the FFQ and 3-day dietary record nutrient intakes. The residual method was used to obtain energy-adjusted data for nutrient correlation [26]. Agreements in quartile classification were calculated to validate the agreement of intake between the FFQ and 3-day dietary records, and the weighted kappa statistic was estimated. All statistical analyses were performed using Statistical Analysis Software (version 9.4, SAS Institute, Cary, NC, USA).

## 3. Results

### 3.1. Development of FFQ

The list of dishes with relation to twenty-one DFs in the contribution and variability analyses is summarized in Table 1. The most frequently selected dishes in the contribution analysis were “Gimbap (rice rolled in laver)” and “Kimchi stew”, which contributed significantly to the intake of a total of ten DFs (Gimbap: energy, protein, fat, Na, Fe, vitamin C, fish, fish2, vegetable, vegetable1; Kimchi stew: protein, fat, Na, Fe, vitamin C, fish, fish2, garlic, red meat, vegetable). The next dishes were “Bibimbap (cooked rice with assorted mixtures)”, “Jajangmyeon (Chinese black bean noodles)”, “Soybean paste soup”, and “Soybean paste stew”, which contributed a total of eight DFs. The most frequently selected dish in the variability analysis was “Bibimbap”, which contributed significantly to the intake of a total of eight DFs: vegetable, vegetable1, vegetable2, energy, carbohydrates, protein, fat, and Fe. The next dishes were “Porridge”, “Jjamppong (Chinese noodle soup)”, “Naengmyeon (buckwheat noodles)”, and “Bean sprout soup”, which contributed to a total of seven DFs.

Table 2 presents the number of dishes selected for each total number of DFs. The number of dishes selected ranged from 1 (alcohol, dairy, grain) to 38 (Na) in the contribution analysis and from 1 (alcohol, garlic, grain) to 54 (Fe) in the variability analysis. Through contribution and variability analyses, 196 foods and dishes were selected and regrouped, resulting in a total of 107 items being finally selected, and honey tea and ginger tea were added to finally select 109 items.

### 3.2. Validation Study

One hundred female patients with cancer participated in the validation study, and the mean age of the participants was 42.5 ± 8.3 years. Mean height, weight, and BMI were 162.5 ± 4.9 cm, 58.4 ± 9.4 kg, and 22.1 ±3.2 kg/m^2^, respectively. Almost all participants had breast cancer (98%) and 77% were married. Over 70% of participants had graduated from university, and 55% were housewives (Table 3).

The means and SDs of nutrient intake estimated using the FFQ and 3-day dietary records are summarized in Table 4. Among the nutrients analyzed, energy, carbohydrates, dietary fiber, thiamine, niacin, and potassium intakes reported by the FFQ were significantly higher than those from the 3-day dietary records (*p* < 0.05).

Table 5 shows the results of unadjusted, energy-adjusted, and age- and energy-adjusted Pearson’s correlation coefficients for the FFQ and the 3-day dietary record. The Pearson’s correlation coefficient of energy intake by FFQ and the 3-day dietary record was 0.46, and the Pearson’s correlation coefficients for age- and energy-adjusted carbohydrate, lipid, and protein levels were 0.40, 0.41, and 0.41, respectively. The largest Pearson’s correlation coefficient for energy intake and age-adjusted intake was 0.54 for potassium. The lowest Pearson’s correlation coefficient for energy intake and age-adjusted intake was 0.20 for iron. All correlation coefficients were statistically significant at *p* < 0.05. Agreement was found in quartile classifications of nutrient intake between the FFQ and 3-day dietary records. The same or adjacent classifications of quartiles between the FFQ and 3-day dietary record ranged from 68% (protein) to 81% (energy, dietary fiber). The weighted kappa values ranged from 0.259 (protein) to 0.512 (vitamin C).

## 4. Discussion

This study aimed to develop an FFQ to evaluate the nutritional status of Korean cancer patients and to assess the validity of the FFQ using 3-day diet records. Patients with cancer should maintain proper nutritional status to overcome cancer, alleviate the side effects of chemotherapy, and prevent recurrence [27,28]. However, nutritional survey tools for evaluating the nutritional status of patients with cancer are limited in Korea. In this study, using the 2016–2018 KNHANES data, we selected dish items to include in the FFQ by considering nutritional contribution and between-person variability of CRDFs using the CA and VA methods. Portion sizes and recipes for the dish items were determined using the 2016–2018 KNHANES data, which are reliable data that reflect the characteristics of Koreans.

The FFQ is a nutritional survey tool that is widely used to determine long-term nutritional status [26]. The European Prospective Investigation into Cancer (EPIC) study, one of the largest cohort studies in Europe, used the FFQ as a dietary assessment tool [29] and the Nurses’ Health Study also used a semi-quantitative FFQ from 1980 [30]. In a German cohort of EPIC, the FFQ had fairly good validity and reproducibility for most nutrients with 24 h dietary recall data [25], and the Netherlands Diet and Cancer cohort also reported that the FFQ correlated well with the overall dietary record [31]. A validation study of adults in Lebanon demonstrated that the FFQ is a suitable method for assessing dietary intake, as it correlates fairly well with nutrient estimates of 24 h dietary recall and is appropriate for evaluating dietary intake in a large population [32]. In addition, the American Cancer Society used the FFQ in the Cancer Prevention Study-3 cohort study to assess the quality of diet in participants according to cancer prevention diet guidelines. Consequently, it was found that FFQ can reliably and effectively evaluate the quality of diets compared to the 24 h dietary recall [11].

The 3-day dietary record method has been widely used as a reference method in several previous studies that analyzed the validity of the FFQ [33,34,35]. In our study, the FFQ tended to slightly overestimate nutrient intake compared to dietary records, especially energy and carbohydrates. However, this overestimation has also been found in previous studies [32,36,37,38,39].

Our FFQ showed age- and energy–adjusted correlations of 0.20 (iron) to 0.54 (potassium) with the 3-day diet records of patients with cancer. This correlation between FFQ and 3-day diet records was relatively higher for most nutrients compared to other FFQs developed with similar methods: energy 0.46 vs. 0.40, carbohydrates 0.40 vs. 0.24, fat 0.41 vs. 0.38, protein 0.41 vs. 0.32, vitamin A 0.49 vs. 0.25, β-carotene 0.44 vs. 0.29, niacin 0.22 vs. 0.36, vitamin C 0.52 vs. 0.30, calcium 0.27 vs. 0.42, sodium 0.29 vs. 0.25, potassium 0.54 vs. 0.27, and iron 0.20 vs. 0.20 [40]. Adjusted Pearson’s correlation coefficients reported in the KNHANES FFQ validation study ranged from 0.15 (thiamin) to 0.64 (carbohydrates) [39].

In our study, the proportion of agreement in same or adjacent quartiles was highest for energy (81%) and fiber (81%), and relatively low for protein (68%). Moreover, the weighted kappa values ranged from 0.259 (protein) to 0.512 (vitamin C), mostly exceeding 0.20. These results were similar to those reported in a study of 305 Korean adults, in which more than 75% of the subjects were classified in the same or adjacent quartiles, and the weighted kappa value ranged from 0.18 to 0.57 [34]. Another Korean study reported an agreement between FFQ and diet records of 64% for nutrients and 65% for food [40]. In a Lebanese study, subjects were classified into the same and adjacent quartiles from 64.3% (polyunsaturated fatty acids) to 83.9% (alcohol) and weighed kappa values ranged from 0.02 (polyunsaturated fatty acids) to 0.36 (energy) [41].

Our study had several limitations. First, since most of the participants were women with breast cancer, it is difficult to generalize the validity results of the FFQ to all patients with cancer. However, breast cancer is the most common cancer among Korean women, excluding thyroid cancer, and is closely related to dietary intake; therefore, nutritional management is required. Second, the number of validation study participants was relatively small (*n* = 100); however, other validation studies have also been conducted with smaller numbers [42,43,44,45,46]. Willet recommended a sample size of >50 individuals and an ideal sample size of 100–200 individuals in an epidemiological study [47]. Third, the 3-day dietary records were collected only once; therefore, it may be difficult to accurately measure seasonal variability. However, daily variability could be minimized with dietary records of 2 days on weekdays and 1 day on the weekend.

The strength of this study is that the FFQ was developed based on a dish-based approach that included ingredients such as cooking oils, seasonings, and spices, enabling more accurate estimates of fat, fatty acid, and sodium intake [34]. Moreover, ginger and honey, known to be effective in alleviating the side effects of chemotherapy, were added to the FFQ list [19,23]. This FFQ could be used to evaluate the effects of dietary factors associated with chemotherapy side effects in patients with cancer.

This FFQ showed good validity with respect to the 3-day dietary records of macro- and micronutrients in patients with cancer. In addition, this FFQ is currently being used in a cohort study investigating the nutritional status and dietary patterns of cancer patients and is expected to be used in various studies to observe dietary factors related to cancer [48,49,50,51]. Therefore, the FFQ developed in this study can be helpful for long-term nutritional status evaluation and management of patients with cancer in Korea, and could be used for epidemiological studies relevant to cancer prognosis.

## 5. Conclusions

In this study, we developed and validated an FFQ for the dietary assessment of patients with cancer considering cancer-related dietary factors. This FFQ showed good validity with respect to the 3-day dietary records in patients with cancer. Therefore, this FFQ may help evaluate the nutritional status of patients with cancer and provide a nutritional guide to those suffering from the side effects of chemotherapy.

## Figures and Tables

**Table 1:** Selected dishes from both the contribution analysis (CA) and variability analysis (VA) for the highest number of cancer- and non-cancer-related dietary factors.

Type of Analysis	Number of Dietary Factor (DF)	Selected Dishes
CA	10	Gimbap (rice rolled in laver), Kimchi stew
	8	Bibimbap (cooked rice with assorted mixtures), Jajangmyeon (Chinese black bean noodles), Soybean paste stew, Soybean paste soup
	6	Multigrain rice, Ramen, Bread, Bean sprout soup
	5	Fried rice, Rice cake, Sandwich, Sea mustard soup
VA	8	Bibimbap
	7	Porridge, Jjamppong (Chinese noodle soup), Naengmyeon (buckwheat noodle), Bean sprout soup
	6	Gimbap, Jajangmyeon
	5	Sushi, Bread, Sandwich, Soybean paste stew, Soybean paste soup

**Table 2:** Number of selected dishes through contribution analysis and variability analysis.

	Number of Selected Dishes		
	By Contribution Analysis (Cumulative Contribution Rate) *	By Variability Analysis (Cumulative R^2^) †	Number of Total Dishes in the FFQ
Energy	19	36	37
Carbohydrates	9	33	34
Protein	37	57	67
Fat	31	19	34
Na	38	49	58
Fe	33	54	58
Vitamin C	20	6	21
Alcohol	1	1	1
Dairy	1	3	3
Fish, total	15	17	23
Fish except for canned fish	11	12	16
Back blue fish including canned fish	5	9	11
Garlic	17	1	17
Grain	1	1	1
Legume	5	4	5
Processed meat	4	4	5
Red meat	7	6	8
Vegetables, total	22	35	37
Vegetables except for kimchi	35	41	46
Raw vegetables	24	27	31
Kimchi	1	4	4
Total	125	185	
Extracted dishes ‡	196		
Regrouped dishes §	107		
Final selected dishes *∥*	109		

* The number of dishes accounting for more than 50% of the cumulative contribution rate for each of the 21 dietary factors (DFs) (17 CRDFs and 4 non-CRDFs); † The number of dishes accounting for more than 70% of inter-individual variability based on the R^2^ for each of the 21 items; ‡ The number of dishes accounting for more than 50% of the cumulative contribution rate or 70% of inter-individual variability based on the R^2^ for each of the 21 items; § The resulting 107 dishes are regrouped by the similarity of cooking method and main ingredients; *∥* By adding honey tea and ginger tea, 109 food items were finally selected.

**Table 3:** General characteristics of participants (*n* = 100).

Variables	Mean	±	SD
Women, *n* (%)	100		(100.0)
Age (years)	42.5	±	8.3
Weight (kg)	58.4	±	9.4
Height (cm)	162.5	±	4.9
BMI (kg/m^2)^	22.1	±	3.2
Type of cancer, *n* (%)			
Breast	98		(98.0)
Colorectal	1		(1.0)
Stomach	1		(1.0)
Current alcohol drinking, *n* (%)			
No	83		(83.0)
Yes	17		(17.0)
Current smoking, *n* (%)			
No	98		(98.0)
Yes	2		(2.0)
Physical activity, *n* (%)			
No	45		(45.0)
Yes	55		(55.0)
Education level, *n* (%)			
Elementary school	1		(1.0)
Middle school	0		(0.0)
High school	20		(20.0)
College and higher	79		(79.0)
Household income, *n* (%)			
<1,000,000 won	6		(6.0)
1,000,000–2,000,000 won	4		(4.0)
2,000,000–4,000,000 won	37		(37.0)
>4,000,000 won	53		(53.0)
Marital status, *n* (%)			
Married	77		(77.0)
Others	23		(23.0)
Job, *n* (%)			
White-collar worker	26		(26.0)
Service worker	5		(5.0)
Blue-collar worker	2		(2.0)
Housewife	55		(55.0)
Others	12		(12.0)

SD: Standard Deviation.

**Table 4:** Comparison of nutrient intake using FFQ and 3-day dietary records (*n* = 100).

	FFQ			3-Day Dietary Records			*p*-Value *
	Mean	±	SD	Mean	±	SD	
Energy (kcal)	1804.8	±	469.4	1552.0	±	349.6	<0.0001
Carbohydrates (g)	268.5	±	71.4	211.9	±	50.7	<0.0001
Fat (g)	48.2	±	18.8	47.7	±	15.7	0.9019
Protein (g)	71.0	±	22.1	67.8	±	20.7	0.2507
Dietary fiber (g)	26.5	±	9.8	22.6	±	7.9	0.0015
Vitamin A (μg RAE)	563.3	±	398.5	494.4	±	374.3	0.1809
Retinol (μg)	161.7	±	87.3	139.4	±	82.4	0.0609
β-carotene (μg)	4820.1	±	4453.0	4260.2	±	4360.4	0.266
Thiamin (mg)	1.9	±	0.6	1.5	±	0.5	<0.0001
Riboflavin (mg)	1.6	±	0.6	1.4	±	0.4	0.0609
Niacin (mg)	15.1	±	4.4	12.8	±	4.1	0.0003
Vitamin C (mg)	140.0	±	64.8	147.2	±	81.6	0.8366
Calcium (mg)	485.3	±	190.5	500.5	±	182.5	0.5525
Sodium (mg)	3161.7	±	1250.9	3297.5	±	1139.5	0.335
Potassium (mg)	3186.5	±	1062.2	2654.0	±	832.7	0.0002
Iron (mg)	16.4	±	6.0	16.4	±	6.1	0.8385

SD: Standard Deviation; * Wilcoxon signed-rank test for nutrient intake between FFQ and 3-day dietary records.

**Table 5:** Correlation coefficients and agreement for nutrient intake between FFQ and 3-day dietary records (*n* = 100).

	Unadjusted Pearson Correlation	*p*-Value	Energy-Adjusted Pearson Correlation	*p*-Value	Age- and Energy-Adjusted Pearson Correlation	*p*-Value	Cross Classification (%)		Weighted Kappa
							Same Quartile	Same or Adjacent Quartile	
Energy (kcal)	0.46	<0.0001					32	81	0.409
Carbohydrates (g)	0.48	<0.0001	0.38	<0.0001	0.40	<0.0001	36	78	0.432
Lipid (g)	0.40	<0.0001	0.40	<0.0001	0.41	<0.0001	35	75	0.388
Protein (g)	0.34	0.0005	0.39	<0.0001	0.41	<0.0001	29	68	0.259
Dietary fiber (g)	0.50	<0.0001	0.51	<0.0001	0.50	<0.0001	30	81	0.392
Vitamin A (μg RAE)	0.45	<0.0001	0.49	<0.0001	0.49	<0.0001	38	71	0.394
Retinol (μg)	0.30	0.0027	0.28	0.0043	0.29	0.0031	31	77	0.367
β-carotene (μg)	0.44	<0.0001	0.47	<0.0001	0.46	<0.0001	34	77	0.388
Thiamin (mg)	0.42	<0.0001	0.37	0.0001	0.37	0.0002	39	73	0.394
Riboflavin (mg)	0.42	<0.0001	0.36	0.0002	0.38	0.0001	33	75	0.369
Niacin (mg)	0.30	0.0024	0.20	0.0485	0.22	0.0289	29	74	0.294
Vitamin C (mg)	0.57	<0.0001	0.52	<0.0001	0.52	<0.0001	45	80	0.512
Calcium (mg)	0.30	0.0024	0.28	0.0055	0.27	0.006	25	76	0.285
Sodium (mg)	0.28	0.005	0.30	0.0027	0.29	0.0038	29	75	0.330
Potassium (mg)	0.50	<0.0001	0.54	<0.0001	0.54	<0.0001	35	76	0.415
Iron (mg)	0.30	0.0027	0.21	0.0355	0.20	0.0468	31	70	0.324

## Data Availability

All data are reported in this manuscript.

## References

[1] Korea National Cancer Center (2019). Annual Report of Cancer Statistics in Korea in 2019.

[2] Jun S., Ha K., Chung S., Joung H. (2016). Meat and milk intake in the rice-based Korean diet: Impact on cancer and metabolic syndrome. Proc. Nutr. Soc..

[3] Clinton S.K., Giovannucci E.L., Hursting S.D. (2020). The World Cancer Research Fund/American Institute for Cancer Research Third Expert Report on Diet, Nutrition, Physical Activity, and Cancer: Impact and Future Directions. J. Nutr..

[4] Mittelman S.D. (2020). The Role of Diet in Cancer Prevention and Chemotherapy Efficacy. Annu. Rev. Nutr..

[5] (2016). Eropean Code Against Cancer.

[6] Stepien M., Chajes V., Romieu I. (2016). The role of diet in cancer: The epidemiologic link. Salud Publica Mex..

[7] Korea National Cancer Center (2015). Estimation of Population Attributable Fraction of Diet for Cancer Prevention and Control in Korea.

[8] Woo H.D., Park S., Oh K., Kim H.J., Shin H.R., Moon H.K., Kim J. (2014). Diet and cancer risk in the Korean population: A meta-analysis. Asian Pac. J. Cancer Prev..

[9] Ko K.P., Park S.K., Yang J.J., Ma S.H., Gwack J., Shin A., Kim Y., Kang D., Chang S.H., Shin H.R. (2013). Intake of soy products and other foods and gastric cancer risk: A prospective study. J. Epidemiol..

[10] Kim S.Y., Wie G.A., Cho Y.A., Kang H.H., Ryu K.A., Yoo M.K., Jun S., Kim S.A., Ha K., Kim J. (2017). The Role of Red Meat and Flavonoid Consumption on Cancer Prevention: The Korean Cancer Screening Examination Cohort. Nutrients.

[11] Troeschel A.N., Hartman T.J., Flanders W.D., Wang Y., Hodge R.A., McCullough L.E., Mitchell D.C., Sampson L., Patel A.V., McCullough M.L. (2020). The American Cancer Society Cancer Prevention Study-3 FFQ Has Reasonable Validity and Reproducibility for Food Groups and a Diet Quality Score. J. Nutr..

[12] Subar A.F. (2004). Developing dietary assessment tools. J. Am. Diet. Assoc..

[13] Willett W.C., Hu F.B. (2007). The food frequency questionnaire. Cancer Epidemiol. Biomark. Prev..

[14] Willett W.C., Hu F.B. (2006). Not the time to abandon the food frequency questionnaire: Point. Cancer Epidemiol. Biomark. Prev..

[15] Anderson P.M., Lalla R.V. (2020). Glutamine for Amelioration of Radiation and Chemotherapy Associated Mucositis during Cancer Therapy. Nutrients.

[16] Park M.K., Kim D.W., Kim J., Park S., Joung H., Song W.O., Paik H.Y. (2011). Development of a dish-based, semi-quantitative FFQ for the Korean diet and cancer research using a database approach. Br. J. Nutr..

[17] Shin W.K., Song S., Hwang E., Moon H.G., Noh D.Y., Lee J.E. (2016). Development of a FFQ for breast cancer survivors in Korea. Br. J. Nutr..

[18] Key T.J., Bradbury K.E., Perez-Cornago A., Sinha R., Tsilidis K.K., Tsugane S. (2020). Diet, nutrition, and cancer risk: What do we know and what is the way forward?. BMJ.

[19] Liu T.M., Luo Y.W., Tam K.W., Lin C.C., Huang T.W. (2019). Prophylactic and therapeutic effects of honey on radiochemotherapy-induced mucositis: A meta-analysis of randomized controlled trials. Support. Care Cancer.

[20] Yang C., Gong G., Jin E., Han X., Zhuo Y., Yang S., Song B., Zhang Y., Piao C. (2019). Topical application of honey in the management of chemo/radiotherapy-induced oral mucositis: A systematic review and network meta-analysis. Int. J. Nurs. Stud..

[21] Cho H.K., Jeong Y.M., Lee H.S., Lee Y.J., Hwang S.H. (2015). Effects of honey on oral mucositis in patients with head and neck cancer: A meta-analysis. Laryngoscope.

[22] An W., Li S., Qin L. (2021). Role of honey in preventing radiation-induced oral mucositis: A meta-analysis of randomized controlled trials. Food Funct..

[23] Chang W.P., Peng Y.X. (2019). Does the Oral Administration of Ginger Reduce Chemotherapy-Induced Nausea and Vomiting?: A Meta-analysis of 10 Randomized Controlled Trials. Cancer Nurs..

[24] Crichton M., Marshall S., Marx W., McCarthy A.L., Isenring E. (2019). Efficacy of Ginger (*Zingiber officinale*) in Ameliorating Chemotherapy-Induced Nausea and Vomiting and Chemotherapy-Related Outcomes: A Systematic Review Update and Meta-Analysis. J. Acad. Nutr. Diet..

[25] Bohlscheid-Thomas S., Hoting I., Boeing H., Wahrendorf J. (1997). Reproducibility and relative validity of energy and macronutrient intake of a food frequency questionnaire developed for the German part of the EPIC project. European Prospective Investigation into Cancer and Nutrition. Int. J. Epidemiol..

[26] Willett W.C., Sampson L., Stampfer M.J., Rosner B., Bain C., Witschi J., Hennekens C.H., Speizer F.E. (1985). Reproducibility and validity of a semiquantitative food frequency questionnaire. Am. J. Epidemiol..

[27] Ravasco P. (2019). Nutrition in cancer patients. J. Clin. Med..

[28] Lee H.-O., Lee J.-J. (2015). Nutritional intervention using nutrition care process in a malnourished patient with chemotherapy side effects. Clin. Nutr. Res..

[29] Kroke A., Klipstein-Grobusch K., Voss S., Möseneder J., Thielecke F., Noack R., Boeing H. (1999). Validation of a self-administered food-frequency questionnaire administered in the European Prospective Investigation into Cancer and Nutrition (EPIC) Study: Comparison of energy, protein, and macronutrient intakes estimated with the doubly labeled water, urinary nitrogen, and repeated 24-h dietary recall methods. Am. J. Clin. Nutr..

[30] Bao Y., Bertoia M.L., Lenart E.B., Stampfer M.J., Willett W.C., Speizer F.E., Chavarro J.E. (2016). Origin, methods, and evolution of the three Nurses’ Health Studies. Am. J. Public Health.

[31] Sturmans F., Hermus R. (1994). Validation of a dietary questionnaire used in a large-scale prospective cohort study on diet and cancer. Eur. J. Clin. Nutr.

[32] Khaled K., Hundley V., Bassil M., Bazzi M., Tsofliou F. (2021). Validation of the European Prospective Investigation into Cancer (EPIC) FFQ for use among adults in Lebanon. Public Health Nutr..

[33] Lee Y., Park K. (2016). Reproducibility and validity of a semi-quantitative FFQ for trace elements. Br. J. Nutr..

[34] Na Y.J., Lee S.H. (2012). Development and validation of a quantitative food frequency questionnaire to assess nutritional status in Korean adults. Nutr. Res. Pract..

[35] Bae Y.-J., Choi H.-Y., Sung M.-K., Kim M.-K., Choi M.-K. (2010). Validity and reproducibility of a food frequency questionnaire to assess dietary nutrients for prevention and management of metabolic syndrome in Korea. Nutr. Res. Pract..

[36] Mumu S.J., Merom D., Ali L., Fahey P.P., Hossain I., Rahman A., Allman-Farinelli M. (2020). Validation of a food frequency questionnaire as a tool for assessing dietary intake in cardiovascular disease research and surveillance in Bangladesh. Nutr. J..

[37] Tenório M., Wanderley T.M., Macedo I.A., Vanderlei A.L.A., Souza B.G., Ataide-Silva T., de Oliveira A.C.M. (2021). Validation and reproducibility of a FFQ focused on pregnant women living in Northeastern Brazil. Public Health Nutr..

[38] Barbieri P., Crivellenti L.C., Nishimura R.Y., Sartorelli D.S. (2015). Validation of a food frequency questionnaire to assess food group intake by pregnant women. J. Hum. Nutr. Diet..

[39] Kim D.W., Song S., Lee J.E., Oh K., Shim J., Kweon S., Paik H.Y., Joung H. (2015). Reproducibility and validity of an FFQ developed for the Korea National Health and Nutrition Examination Survey (KNHANES). Public Health Nutr..

[40] Park M.K., Noh H.Y., Song N.Y., Paik H.Y., Park S., Joung H., Song W.O., Kim J. (2012). Validity and reliability of a dish-based, semi-quantitative food frequency questionnaire for Korean diet and cancer research. Asian Pac. J. Cancer Prev..

[41] Harmouche-Karaki M., Mahfouz M., Obeyd J., Salameh P., Mahfouz Y., Helou K. (2020). Development and validation of a quantitative food frequency questionnaire to assess dietary intake among Lebanese adults. Nutr. J..

[42] Pakseresht M., Sharma S., Cao X., Harris R., Caberto C., Wilkens L.R., Hennis A.J., Wu S.Y., Nemesure B., Leske M.C. (2011). Validation of a quantitative FFQ for the Barbados National Cancer Study. Public Health Nutr..

[43] Pakseresht M., Miyajima N.T., Shelton A., Iwasaki M., Tsugane S., Le Marchand L., Sharma S. (2013). Validation of a quantitative FFQ for a study of diet and risk of colorectal adenoma among Japanese Brazilians. Public Health Nutr..

[44] Navarro A., Osella A.R., Guerra V., Muñoz S.E., Lantieri M.J., Eynard A.R. (2001). Reproducibility and validity of a food-frequency questionnaire in assessing dietary intakes and food habits in epidemiological cancer studies in Argentina. J. Exp. Clin. Cancer Res..

[45] Henriksen H.B., Carlsen M.H., Paur I., Berntsen S., Bøhn S.K., Skjetne A.J., Kværner A.S., Henriksen C., Andersen L.F., Smeland S. (2018). Relative validity of a short food frequency questionnaire assessing adherence to the Norwegian dietary guidelines among colorectal cancer patients. Food Nutr. Res..

[46] Zhang C.X., Ho S.C. (2009). Validity and reproducibility of a food frequency Questionnaire among Chinese women in Guangdong province. Asia Pac. J. Clin. Nutr..

[47] Willett W. (1998). Nutritional Epidemiology 2.

[48] Krusinska B., Hawrysz I., Wadolowska L., Slowinska M.A., Biernacki M., Czerwinska A., Golota J.J. (2018). Associations of Mediterranean Diet and a Posteriori Derived Dietary Patterns with Breast and Lung Cancer Risk: A Case-Control Study. Nutrients.

[49] Orlich M.J., Mashchak A.D., Jaceldo-Siegl K., Utt J.T., Knutsen S.F., Sveen L.E., Fraser G.E. (2022). Dairy foods, calcium intakes, and risk of incident prostate cancer in Adventist Health Study-2. Am. J. Clin. Nutr..

[50] Xu X., Hendryx M., Liang X., Kahe K., Li Y., Luo J. (2022). Dietary selenium intake and thyroid cancer risk in postmenopausal women. Nutrition.

[51] Pishdad S., Joola P., Bourbour F., Rastgoo S., Majidi N., Gholamalizadeh M., Ebrahimi K., Abbas Torki S., Akbari M.E., Montazeri F. (2021). Association between different types of dietary carbohydrate and breast cancer. Clin. Nutr. ESPEN.

